# Immune cell trafficking: a novel perspective on the gut-skin axis

**DOI:** 10.1186/s41232-024-00334-5

**Published:** 2024-04-24

**Authors:** Jiayan Zhang, Zhirong Yao

**Affiliations:** 1 Dermatology Center, Xinhua Hospital, Shanghai Jiaotong University School of Medicine, Shanghai, China; 2 Department of Dermatology, Xinhua Hospital, Shanghai Jiaotong University School of Medicine, Shanghai, China; 3 Institute of Dermatology, Shanghai Jiaotong University School of Medicine, Shanghai, China

**Keywords:** Gut-skin axis, Immune cell trafficking, Inflammatory diseases

## Abstract

Immune cell trafficking, an essential mechanism for maintaining immunological homeostasis and mounting effective responses to infections, operates under a stringent regulatory framework. Recent advances have shed light on the perturbation of cell migration patterns, highlighting how such disturbances can propagate inflammatory diseases from their origin to distal organs. This review collates and discusses current evidence that demonstrates atypical communication between the gut and skin, which are conventionally viewed as distinct immunological spheres, in the milieu of inflammation. We focus on the aberrant, reciprocal translocation of immune cells along the gut-skin axis as a pivotal factor linking intestinal and dermatological inflammatory conditions. Recognizing that the translation of these findings into clinical practices is nascent, we suggest that therapeutic strategies aimed at modulating the axis may offer substantial benefits in mitigating the widespread impact of inflammatory diseases.

## Background

The skin and gut constitute the two largest immune systems in the human body and employ distinct defense strategies. However, inflammatory diseases frequently co-occur in these sites. Patients with inflammatory bowel disease (IBD) are known to have an increased risk of inflammatory skin disorders [1–3]; conversely, individuals with primary dermatologic conditions exhibit increased susceptibility to IBD [4–6]. While a shared genetic predisposition may signal an increased risk for concurrent skin and gastrointestinal diseases [7], biological communication between the gut and skin is another plausible explanation.

Recent studies have highlighted this bidirectional influence [8, 9]. Specifically, metabolites derived from the gut microbiome such as short-chain fatty acids [10], bile acids [11], and vitamins, as well as neurotransmitters and hormones originating from the gut [12–16], circulate through the blood and affect skin barrier function. Conversely, the skin can produce soluble factors that impact gut health. For instance, compromised skin may release inflammatory cytokines or metabolites that induce or exacerbate gastrointestinal inflammation [17–19].

Beyond the transport of biomolecules through the bloodstream, mounting evidence suggests that the migration of immune cells between the skin and gut during states of immunological imbalance might be a conduit for the spread of pathological conditions and the resultant tissue damage [20, 21]. In fact, the critical role of cell trafficking in the pathogenesis of various diseases has gained increasing recognition in recent years. For example, the development of pathogenic Th17 cells in the small intestine, driven by long-chain fatty acids, has been implicated in disease progression in animal models of central nervous system-mediated inflammation [20]. It has been proposed that complications of IBD such as primary sclerosing cholangitis (PSC) may be mediated by gut-educated lymphocytes, which are recruited in response to the aberrant expression of gut-homing molecules, including MAdCAM-1 and CCL25, within the liver [22, 23]. Similarly, an active homing axis between the gut and inflamed joints has been reported in patients with ankylosing spondylitis, characterized by cells expressing the α_4_β_7_ integrin in inflamed joints and the upregulation of MadCAM-1 in the endothelium [24]. However, while the ectopic expression of gut-associated addressins in skin is an attractive hypothesis for cutaneous extraintestinal manifestations (cEIM), direct evidence in the skin is lacking [25]. The questions of whether abnormal migration of immune cells between the gut and skin can account for the comorbidity of gut and skin inflammation, and what mechanisms underlie such aberrant cell trafficking along the gut-skin axis, remain to be elucidated.

This review delineates the molecular mechanisms of cell migration, the immune architectures of the gut and skin, and the established mechanisms of tissue-specific leukocyte imprinting. It further compiles evidence of immune cell trafficking between the gut and skin in disease states, dissects the underlying mechanisms, and assesses the implications for therapeutic intervention.

## Immune cell trafficking

Cell trafficking plays pivotal roles in both the host defense against pathogens and the establishment of immune tolerance. By migrating to specialized microenvironments, immune cells experience functional modulation to shape local immunity and influence inflammatory responses [26]. The intricate process of trafficking encompasses various types of movements, such as homing, retention, recirculation, and amoeboid movement [27, 28]. Although these processes are defined separately, the transitions between them are flexible in a living organism.

### Homing

**Fig. 1:**
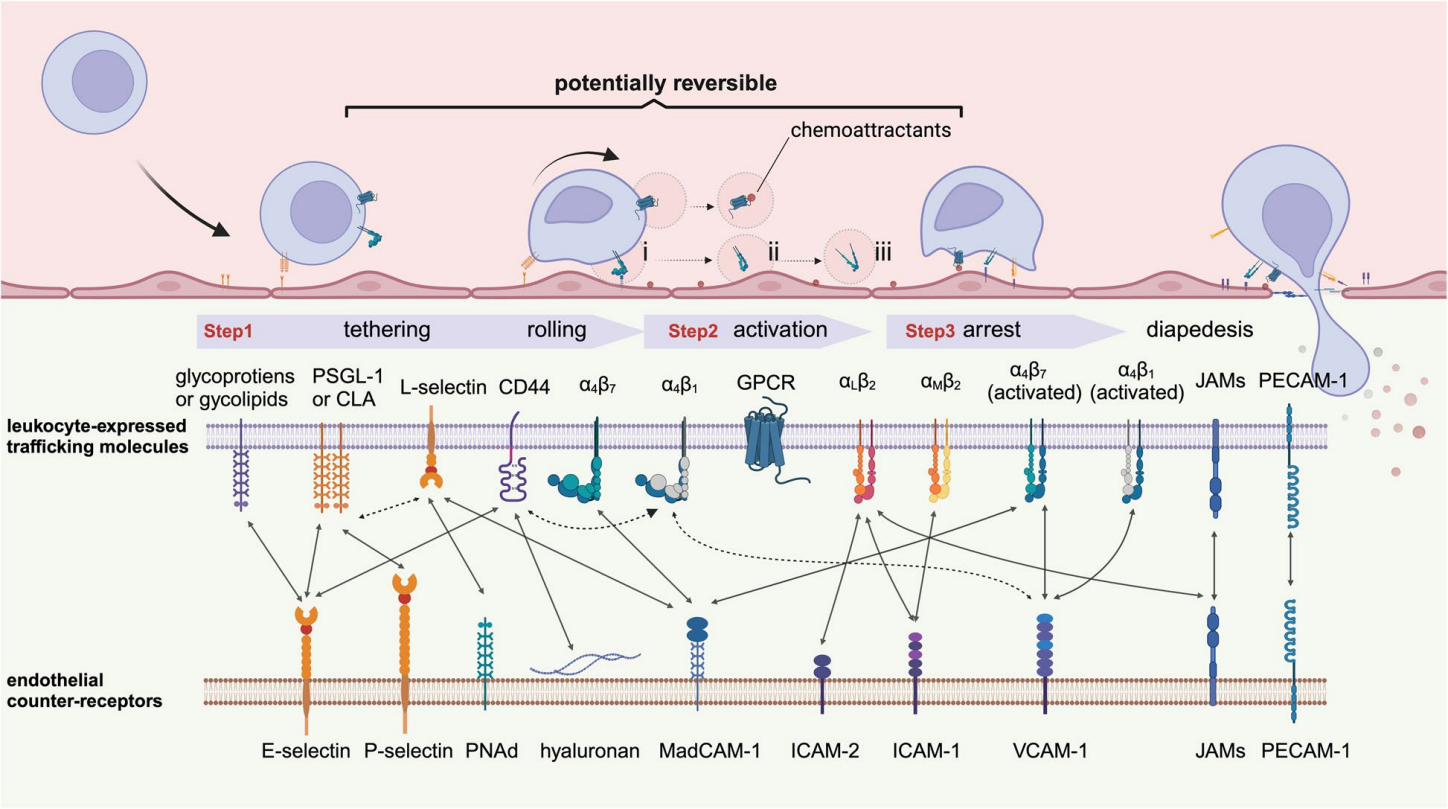
Multistep homing process of lymphocytes. The top panel depicts the multistep adhesion cascade involved in lymphocyte homing: step 1: tethering and rolling. Tethering is mediated primarily by selectins, which can engage with their ligands rapidly with high tensile strength, enabling the capture of leukocytes out of the bloodstream. Integrins facilitate leukocytes’ slower rolling along endothelial cells following capture [37–39]. The adhesive interactions in this step are notably reversible and transient. Step 2: integrin activation. External stimuli are required for integrin affinity modulation [40]. Integrins have three conformational states with differing affinities [40]: (i) bent head-piece conformation with low affinity, (ii) extended head-piece conformation with intermediate affinity, and (iii) extended head-piece conformation with high affinity. Specific endothelial chemokines can rapidly (within milliseconds) enhance integrin affinity [41]. Step 3: arrest. This step is mediated by activated integrins, whereby lymphocytes adhere firmly to the endothelium and come to a complete stop. The bottom of the diagram shows the predominant molecules expressed on lymphocytes and endothelial cells involved at each step [27, 29, 42].. The arrows between them represent potential interactions (broken arrows indicate weak binding)

Immune cells are equipped with programmed homing receptors designed to identify tissue-specific adhesion molecules and chemoattractants, thus ensuring the timely allocation of immune effector mechanisms [29]. Homing follows a multistep adhesion cascade (Fig. 1). Initially, selectins and integrins on the surface of immune cells engage with their respective ligands on the vascular endothelium, mediating “tethering” and “rolling” of leukocytes along the vessel wall. Subsequently, endothelial-presented chemokines activate G protein-coupled receptors on leukocytes [30, 31], triggering integrin β subunit cytoplasmic tails to bind with talin [32]. Such binding induces conformational changes of the integrin extracellular domains into high-affinity, extended forms [33–35], leading to the firm “arrest” of cells on the luminal face of postcapillary venules. The leukocytes then navigate through the endothelial junctions, propelled by shear forces, adhesive interactions, and chemoattractant gradients, finally disseminating into designated microenvironmental niches [36].

### Retention

Immune cells can be resident in tissues, and this localization is integral to their role in innate and adaptive responses. Innate immune cells are seeded directly into tissues following their development during early embryonic and fetal stages. This process is orchestrated by distinct waves of hematopoiesis that give rise to specialized precursor cells, which subsequently seed developing tissues and acquire tissue-specific phenotypes [43, 44]. In contrast, lymphocytes, the cellular components of adaptive immunity, can take up residence in peripheral tissues following antigen encounter. Upon tissue entry, these cells, including tissue-resident memory T and B cells, coordinate antipathogen immunity and provide long-term protection against future infectious challenges [45].

The retention of immune cells in tissues is mediated by the upregulation of specific markers. Key markers of tissue-resident lymphocytes include CD69, CD103 (α_E_β_7_), CD49a, and CD49b. CD69, serving as an endogenous negative regulator, interacts with sphingosine 1-phosphate receptor 1 (S1PR1) in cis to mediate internalization and degradation of this receptor [46]. Given that the chemotactic ligand for S1PR1, sphingosine 1-phosphate (S1P), is abundant in blood and lymph but scant in tissues, CD69 impedes the egress of cells following the S1P gradient [47, 48]. CD103 binds E-cadherin on epithelial cells, thereby promoting cell accumulation in intestinal, cutaneous, bronchial, and genital epithelia [49, 50]. CD49a and CD49b are collagen-binding integrins that primarily adhere to type IV and I collagen [51–53]. Other molecules, such as antigen-specific T cell receptors [54, 55] and some chemokine receptors [56–58] (e.g., CXCR4 and CCR7), are also implicated in cell retention through recognition of cognate antigens or constitutively expressed chemotactic ligands by structural cells [57]. Collectively, immune cells enhance their retention capacity upon inflammatory cytokine response [59] or T cell receptor stimulation, thereby performing tissue-specific functions in immunity [45, 60].

### Recirculation and amoeboid migration

Immune cell recirculation connects various microenvironments and involves a range of cells, including dendritic cells (DCs), lymphocytes, monocytes, and granulocytes. These cells migrate through the afferent lymphatics from non-lymphoid tissues into draining lymph nodes and may then travel to adjacent lymph nodes. The detailed mechanisms of their entry and migration within afferent lymphatics have been thoroughly reviewed elsewhere [61]. Furthermore, some of these cells can re-enter the bloodstream through an S1P-driven mechanism [62, 63].

The locomotion of immune cells within tissues is orchestrated by amoeboid migration, a multiscale phenomenon coupling cell shape changes, biochemical signaling, and cytosolic and extracellular fluid flows [64, 65].

## The immunological anatomy of the skin and intestinal mucosa

### Skin immune system

**Fig. 2:**
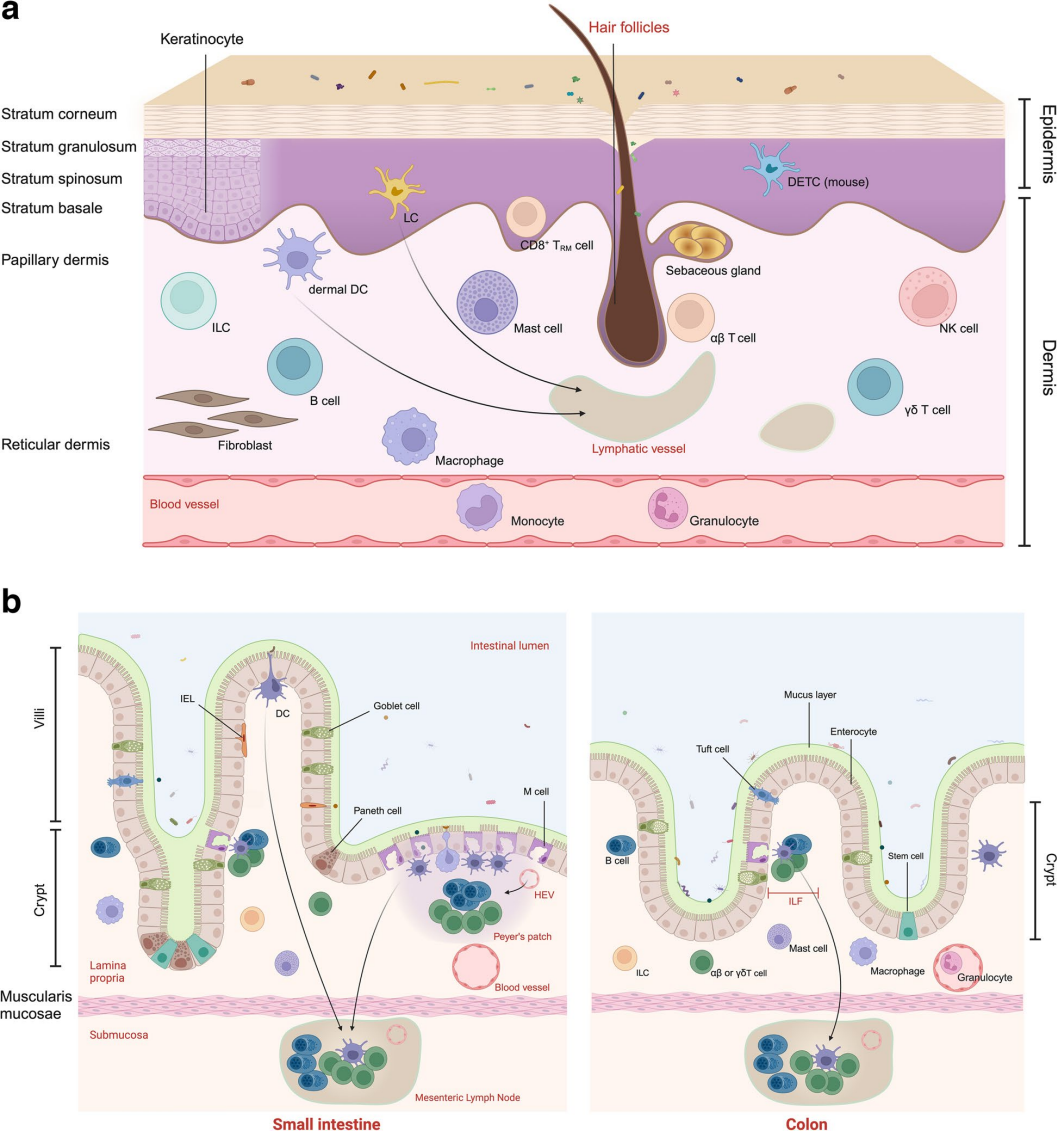
**a** The structure and immune cell distribution of the skin under steady state. The epidermis represents the outermost layer of the skin. The dermis can be divided into the superficial papillary layer and the deeper reticular layer. Blood and lymphatic vessels, as well as nerves (not shown), pervade the dermis. Under normal conditions, the most common immune cells in human epidermis are LCs, located in the stratum spinosum, and CD8^+^ tissue-resident memory T (TRM) cells, found in the stratum basale and stratum spinosum [70]. CD8^+^ TRM cells can migrate between the epidermis and the papillary dermis, performing tissue patrols [71]. The mouse epidermis contains dendritic epidermal T cells (DETCs), a cell type absent in humans. In healthy skin, dermal leukocytes encompass DCs, macrophages, mast cells, γδ T cells, natural killer (NK) cells, innate lymphoid cells (ILCs), αβ T cells, and B cells. Most αβ T cells in the dermis are CD4^+^ T cells, while B cells are rarely present in normal skin [72]. Skin appendages include hair follicles, sebaceous glands, and sweat glands (not shown). Commensal microorganisms inhabit the epidermis, dermis, and dermal appendages, forming an additional layer of host defense [73]. **b** Epithelial composition and immune cell distribution of the intestine under steady state. The intestinal epithelium is composed of a single layer of cells, arrayed into projections known as villi which extend into the intestinal lumen, and moat-like invaginations called crypts that surround the villi. Multipotent stem cells are located at the base of these crypts, interspersed among Paneth cells. These stem cells have the capability to differentiate into intestinal absorptive cells and all types of specialized epithelial cells, including goblet cells, Paneth cells, microfold-cells (M-cells), and tuft cells [74]. Intraepithelial lymphocytes (IELs) display high levels of activity. They are typically situated between the basement membrane and the epithelial layer of the intestinal villi under steady-state conditions, occasionally demonstrating transient movements closely associated with epithelial cells [75]. Peyer’s patches (PPs) are unique tertiary lymphoid organs in the small intestine, and isolated lymphoid follicles (ILFs) are distributed along the length of both the small and large intestines. Compared to the small intestine, the colonic epithelium lacks villi structures and IELs are rarely observed. Paneth cells are typically found only in the small intestine, but are present in the colon during inflammatory conditions. The lamina propria is composed of loose connective tissue traversed by blood vessels, lymphatic vessels, and nerves (not shown), and houses numerous innate and adaptive immune cells. M-cells, macrophages, and dendritic cells (DCs) are responsible for sampling antigens and triggering specific T cell and B cell responses within GALT

The skin consists of the epidermis and dermis, demarcated by the basement membrane zone. The epidermis is predominantly composed of keratinocytes, supplemented by a minor fraction of T cells and Langerhans cells (LCs) [66]. Depending on the degree of keratinocyte differentiation, the epidermis can be stratified from the outermost to the innermost layer into the stratum corneum, granulosum, stratum spinosum, and stratum basale (Fig. 2a). The stratum corneum is the epidermis’s most unique anatomical feature and serves as a barrier that impedes the permeation of water and water-soluble substances, as well as prevents the entry of external pathogens. Beyond their role as a physical barrier, keratinocytes are also instrumental in recruiting immune cells to the epithelial interface, where they regulate cell survival and retention [67]. LCs are professional antigen-presenting cells (APCs) embedded among keratinocytes [68]. Upon activation, they downregulate E-cadherin and upregulate CCR7 to migrate to skin-draining lymph nodes and prime naive T cells, initiating immune responses that may either induce immunological tolerance or spur the expansion of pro-inflammatory effector and memory T cell populations [69].

The dermis, consisting of fibroblasts and connective tissue, provides essential structural support to the skin [76]. Cells traverse to and from the skin via vascular and lymphatic vessels in the dermis, resulting in a higher cellular diversity in the dermal compartment compared to the epidermis (Fig. 2a) [77–79]. Following skin immunization, dermal dendritic cells (dDCs) migrate to lymph nodes faster than LCs [69], and certain subsets of dDCs tend to move into the outer paracortex to regulate the differentiation of B cells [80, 81]. In healthy skin, dermal macrophages remain unable to migrate to draining lymph nodes.

Skin appendages, which extend from the epidermis into the dermis, provide critical niches for microbial colonization and transdermal penetration of various compounds due to their invaginated architecture and absence of a stratum corneum [82]. They also represent unique immunological sites. Hair follicles offer protection to LCs from environmental damage, such as ultraviolet radiation, thus preserving a reservoir of APCs [83]. Sebaceous and sweat glands contribute to the immune response by secreting chemokines, cytokines, and antimicrobial peptides [84, 85].

### Intestinal immune system

In contrast to the skin, the gastrointestinal tract lacks a stratum corneum and is therefore more permeable. As a result, it is continuously exposed to antigens and immune modulators derived from diet and the microbiome. Functionally, the intestinal immune compartment can be divided into inductive sites and effector sites, the former being gut-associated lymphoid tissue (GALT) and the latter encompassing the epithelium and lamina propria [86]. Among the intestinal epithelial cells reside intraepithelial lymphocytes (IELs). The immune cells present in the lamina propria include DCs, macrophages, mast cells, granulocytes, NK cells, γδ T cells, innate lymphoid cells (ILCs), αβ T cells, and B cells [87, 88]. Macrophages in the gastrointestinal tract exhibit a remarkable functional diversity, adapted to the specific ecological niches they inhabit. They are distributed among neurons, blood vessels, Peyer’s patches (PPs), crypts, and the epithelium. Notably, macrophages residing in the lamina propria possess high phagocytic capacity and can present antigens to DCs to induce oral tolerance [89]. In humans, GALT consists primarily of PPs, isolated lymphoid follicles (ILFs), MLNs, and lymphoid tissues in the appendix and rectum. T or B cells activated within PPs can migrate to MLNs for further proliferation and differentiation [90, 91].

Strictly speaking, the mucosa does not include MLNs as it consists of the epithelium, lamina propria, and muscularis mucosae (Fig. 2b). However, MLNs play a pivotal role in mucosal immune responses (as detailed later). Lamina propria DCs (lpDCs) sample lumen antigens without disrupting tight junctions and migrate to the MLNs by upregulating CCR7, where they prime naive T cells to differentiate into regulatory or effector subsets [92]. Interestingly, not only lpDCs but also some DCs from PPs and ILFs migrate to MLNs in a CCR7-dependent manner. Yet, under steady-state conditions, DCs do not exit from MLNs into efferent lymphatics [93, 94]. In all, the trafficking of immune cells from mucosal inductive to effector tissues via the lymphatic system forms the cellular foundation for the immune response in the gastrointestinal tract [95].

## Immune cell trafficking along the gut-skin axes

### Tissue-specific imprinting of leukocytes homing

Prevailing dogma holds that priming of immune cells in specific inductive regions leads to distinct homing programs biased towards trafficking to associated tissues and microenvironments [96]. There is a hypothesis that each tissue possesses unique “area codes” [62] comprising not only specific combinations of adhesion molecules and chemokines [97], but also environmental cues from sources like food (vitamin A) and sunlight (vitamin D3) in the case of gut and skin, respectively [98].

Retinoic acid (RA), a metabolite of vitamin A, can be produced by intestinal DCs and stromal cells, as well as by the intestinal bacteria [99–101]. When T cells and B cells are activated within GALT, high levels of RA upregulate their expression of gut-homing receptors—α_4_β_7_ integrin and CCR9—while concurrently inhibiting the expression of skin-homing receptors, including selectin ligands [102, 103]. MadCAM-1 and CCL25, the ligands for α_4_β_7_ integrin and CCR9, respectively, are almost explicitly expressed in the small intestine and colon [104] (Table 1). Recent studies have revealed that some innate immune cells, including innate lymphoid cells (ILCs) subsets 1 and 3, along with non-classical monocytes [105, 106], can undergo receptor switching that enhances the expression of α_4_β_7_ integrin and/or CCR9, through mechanisms like those observed in lymphocytes, thereby acquiring gut-homing phenotypes. It is worth noting that, although the effects of RA are often studied in the context of the gut, RA-producing DCs are not confined to this organ. Specific subsets of DCs derived from extraintestinal barrier tissues also express RA under homeostatic conditions, a feature that corresponds with their ecological niches [107].

**Table 1:** Main axes involved in trafficking to skin and gut during health and disease

	**Skin**		**Gut**		
	**Chemokine pathways**	**Adhesion pathways**		**Chemokine pathways**	**Adhesion pathways**
**Increased during inflammation**					
	CCR2–CCL2 [108] CCR4–CCL17/CCL22 [109] CCR5–CCL5 CCR6–CCL20 CCR8–CCL18 CCR10–CCL27 ?–CCL28 [110] CXCR1/2–CXCL8 [111, 112] CXCR2–CXCL1 CXCR3–CXCL9/CXCL10/CXCL11 [113, 114] CXCR4–CXCL12 CXCR5–CXCL13 CXCR6–CXCL16 [114] CX3CR1–CX3CL1	CLA–E selectin/P selectin CD44–E selectin [115] CD44–hyaluronan L-selectin–PNAd α_4_β_1_–VCAM-1 [116] α_L_β_2_–ICAM-1 [117] α_M_β_2_–ICAM-2 PECAM-1 (CD31) GPR15–GPR15L	Small intestine	CCR2–CCL2/7/8 CCR6–CCL20 CXCR3–CXCL9/10/11 CCR5–CCL3/4/5/8 CX3CR1–CX3CL1	α_4_β_7_/L-selectin–MAdCAM-1 α_4_β_1_–VCAM-1 α_L_β_2_–ICAM-1 VAP-1 E-selectin L-selectin–PNAd PSGL-1–P-selectin
			Colon	CCR2–CCL2/7/8 CCR3–CCL11 CCR4–CCL17 CCR6–CCL20 CCR9–CCL25 CXCR1–CXCL5/6/8 CXCR2–CXCL1/2/5/6/8 CXCR3–CXCL9/10/11	α_4_β_7_–MAdCAM-1 [118] L-selectin–PNAd E-selectin
**Constitutive expression**					
	CCR4–CCL17 [119]/CCL22 [119–121] CCR6–CCL20 [122, 123] CCR8–CCL1 [124] CCR10–CCL27 [125]	CD103–E-cadherin CD69 E selectin [72]	Small intestine	CCR6–CCL20 CCR9–CCL25 CXCR1–CXCL5/6/8 CXCR2–CXCL1/2/5/6 CXCR6–CXCL16 CX3CR1–CX3CL1 CXCR5–BCA-1 (CXCL13)	α_4_β_7_–MadCAM-1
			Colon	CCR5–CCL3/4/5/8 CCR6–CCL20 CCR10–CCL28 CX3CR1–CX3CL1 GPR15–GPR15L CXCR5–BCA-1 (CXCL13)	α_4_β_7_–MadCAM-1 CD103–E-cadherin

Table is modified from reference [90, 126]

The situation for skin-homing leukocytes is more complicated. In humans, vitamin D3 is primarily produced by KCs and fibroblasts in sun-exposed skin [127, 128], and is further metabolized into 1,25-dihydroxyvitamin D3 (1,25(OH)_2_D_3_) by KCs, DCs, and macrophages [129]. 1,25(OH)_2_D_3_ has been demonstrated to induce CCR10 expression on antigen experienced T cells [125]. However, other skin-homing receptors, such as CCR8 and CLA, are not induced by 1,25(OH)_2_D_3_ but by soluble mediators from KCs during the activation of naive T cells [130]. Interestingly, factors that induce CLA and CCR10, like IL-12 or 1,25(OH)_2_D_3_, respectively, can inhibit CCR4 and CCR8 expression [131, 132]. The differential regulation by distinct tissue imprinting factors likely reflects the differing homing requirements of specific lymphocytes subsets, either at steady state or during immune activation [133, 134]. Besides T cells, the expression of skin-homing receptors on human B lymphocytes may also depend on the site of antigenic stimulation [135, 136].

### Plasticity in immune cell trafficking along the gut-skin axis

The above-depicted model of issue-specific homing was never meant to be rigid, acknowledging that immune cells can migrate between the gut and skin. The adaptability of this process is partly due to APCs, which modulate the tissue-specific imprinting on immune cells.

The mucosal imprinting capacity of DCs is influenced by the local tissue environment [137]. Under homeostatic conditions, RA signaling promotes gut homing by inducing the expression of ITGA4 and CCR9, which encode the α subunit of α_4_β_7_ integrin and CCR9, respectively [138]. Simultaneously, RA suppresses FUT7, a gene that controls CLA/sLe^X^ expression, which is critical for skin homing. However, this pattern of induction may be disrupted in the presence of inflammatory mediators. Soluble factors from intestinal DCs such as cytokines IL-12/23 may override the suppressive effect of RA on FUT7. These cytokines also enhance the expression of the enzyme C2GlcNAcT-I, pivotal for the formation of P-selectin ligands [139, 140], thereby favoring skin homing [141]. This switch in homing preferences is evidenced by a substantial proportion of activated gut CD4^+^ T cells expressing both skin and gut homing receptors, as seen in animal models of ileitis or colitis and in cases of human Crohn’s disease [141, 142]. These activated gut CD4^+^ T cells are capable of bidirectional migration between the gut and the skin. Furthermore, antigen dose and strength influence homing receptor expression; high antigen doses presented by murine MLN DCs have been shown to reduce α_4_β_7_ and CCR9 expression on effector T cells, curtailing gut infiltration and promoting skin homing [143, 144]. Currently, it is unclear whether the induction of gut homing receptors is effective under inflammatory conditions in the cutaneous environment.

Immune cell trafficking is key in managing disseminated infections, whereby migratory T and B cells can alter their tissue tropism after interacting with tissue-specific DCs and microenvironments [145]. In vitro experiments show that effector-memory T cells initially programmed for gut homing can switch to skin tropism after activation by skin-derived DCs, with corresponding changes in homing receptor expression [146]. Conversely, skin-homing memory T cells can acquire gut homing capabilities following stimulation by intestinal DCs [147]. This flexibility is confirmed in vivo; for example, a subset of CD8^+^ T cells post-skin infection reorient to express gut-homing molecules after migrating to the MLNs. Preventing their early skin-draining lymph nodes egress with FTY720 (an antagonist of S1PR1) hampers this reprogramming [145]. Additionally, unconventional T cells (UTCs) such as γδ T cells and MR1 or CD1d-restricted T cells [148] may also modulate homing patterns [149]. After migrating to draining lymph nodes, UTCs and DCs contribute to site-specific immunity and prepare the immune system for potential future pathogen encounters [70]. It is notable that effector T cells can also localize to non-infected tissues, suggesting other, as yet unidentified, recruitment mechanisms [150].

It is essential to note the pleiotropy and redundancy inherent in cell trafficking pathways [28] (Table 1). For instance, during intestinal inflammation, the recruitment of inflammatory cells mediated by the non-gut-specific homing receptor α_4_β_1_ is crucial for the progression of colitis [151, 152]; its ligand, VCAM-1, is ubiquitously expressed in inflamed tissues [153], which may mediate leukocyte homing to extraintestinal organs.

In summary, immune cells typically exhibit controlled migration patterns, guided by a range of molecules [97]. However, immune dysregulation can lead to aberrant cell migration, potentially exacerbating disease spread. Deviations can occur due to (1) altered imprinting preferences for homing characteristics at their site of activation, (2) reprogramming of homing receptors following encounters with DCs from other tissues, or (3) upregulated expression of gut-specific, skin-specific, or non-specific trafficking molecules by distinct organs. These changes may enable pathogenic immune cells to access distant organs, thus playing a role in disease progression and associated complications [23, 25, 28].

## Aberrant immune cell trafficking between the gut and skin

### Aberrant skin trafficking of gut-derived cells

Recent studies have provided compelling evidence that aberrant trafficking of gut-derived immune cells to the skin contributes to skin inflammation. Omenn syndrome (OS) is an immunodeficiency disorder characterized by early onset erythroderma, enteritis, and tissue infiltration by overactive T cells [154]. Research indicates that using dextran sulfate sodium (DSS) to aggravate colitis in OS mouse models leads to amplified skin inflammation, with a notable increase in circulating CD4^+^ T cells expressing both skin- and gut-homing receptors (CCR4 and CCR9) [155]. Concomitantly, serum lipopolysaccharide binding protein (LBP) levels increase, signaling more systemic antigen translocation and inflammation [155, 156]. However, acute systemic inflammation alone does not trigger skin-specific responses, suggesting that a “leaky gut” is key to exacerbating skin inflammation [155, 157]. Further research by Merana et al. implies that gut inflammation can disrupt the skin’s adaptive immune tolerance to its normal microbial inhabitants. Typically, the immune systems in the gut and skin operate independently, and fluctuations in the gut microbiota do not directly affect cutaneous immune homeostasis [157]. However, this separation can weaken in cases of inflammation [158, 159]. Experiments have shown that colitis prompts a migration of gut-microbe-responsive CD4^+^ T cells to skin-associated lymph nodes, increasing skin neutrophils and decreasing Tregs specific to skin microbes, all of which contribute to skin inflammation [160]. Blocking the travel of lymphocytes can re-establish skin immune tolerance, pointing to the gut as the origin of effector cells in skin inflammation. Classon et al.’s findings agree, demonstrated that treating mice with FTY720, which obstructs cell migration from the gut to the skin, resulted in a decreased presence of skin-directed helminth-specific Th2 CD4^+^ T cells in the context of intestinal helminth infections [161]. Researchers have noted that T cells, which are specific for gut-derived antigens and have migrated from the gastrointestinal tract, are key mediators of skin inflammation. Upon adoptive transfer to naive recipients, these T cells can provoke skin inflammatory responses that are clinically similar to the donor’s condition. Conversely, strategies that impede the trafficking of these effector cells—through genetic manipulation or by antagonizing homing receptors—effectively mitigate the inflammation [157, 162].

The reprogramming of homing receptors, a process that takes place in lymph nodes, is currently known to be the primary mechanism of aberrant migration from the gut to skin. Skin-derived DCs that migrate to peripheral lymph nodes are a potential trigger for this reprogramming [155, 162]. Oyoshi et al.’s pivotal study on AD in mice described a mechanism where antigen-specific intestinal homing CD4^+^ α_4_β_7_^+^ CCR4^−^ T cells, primed via oral allergens, undergo reprogramming in mesenteric or peripheral lymph nodes after encountering skin antigens [162]. These cells then migrate to compromised skin sites in a CCR4-dependent manner. Key evidence includes the presence of skin-derived, antigen-bearing DCs in the MLNs following a cutaneous antigen challenge [162]. Additionally, vascular remodeling and enhanced lymphatic clearance appear to influence the peripheral transportation of skin-derived DCs [155]. The production of vitamin D3 by DCs, upregulated by mechanical skin disruptions like scratching in AD, may also play a role in reprogramming [15, 162].

Contrasting the outlined hypothesis, a study utilizing AD mouse models found unique outcomes. T cells, activated via skin or gut by ovalbumin (OVA), were transferred to naive mice followed by OVA skin challenge. Surprisingly, only the cutaneously activated T cells induced AD-like inflammation [163]. This suggests that antigen presentation specifics, such as dose and exposure duration, are critical for effective reprogramming of homing receptors.

Some studies aimed at optimizing vaccination strategies have also provided evidence for the migration of DCs. Transcutaneous immunization (TCI) has proven effective in generating both systemic and mucosal antibody responses, as well as mucosal cytotoxic T lymphocytes (CTL) responses [164], as evidenced in animal models and human trials [165, 166]. One investigation revealed that following TCI with tetanus toxoid (TT) and adjuvant in mice, a substantial number of TT-specific antibody-secreting cells can be detected in the small intestine and colon [167]. Research has identified that the MLNs serve as the inductive sites for intestinal IgA responses following TCI [168]. Another study reported that TCI with an HIV peptide and adjuvant in mice induced HIV-specific CTLs in the gut-associated lymphoid tissue, conferring protection against mucosal viral challenges. Subsequent experiments suggested possible migration of activated DCs carrying skin-derived antigens from the skin to immune-inductive sites within the gut mucosa [164]. Collectively, these findings suggest that the MLN occupies a key position in immune anatomy, bridging the gut and systemic immune systems.

### Aberrant gut trafficking of skin-derived cells

Pathogenic immune cells can also traffic aberrantly from the skin to the gut, and the mechanisms of their migration appear to differ from those originating in the gut. Emerging research challenges the notion that tissue-resident memory T cells (TRMs) are confined to their tissue of origin [169]. Instead, these cells may recirculate and contribute to systemic inflammatory conditions like IBD [170–175]. Strobl et al.’s study utilizing allogeneic hematopoietic stem cell transplantation (HSCT) as a model uncovered that patients with active graft-versus-host disease (GVHD) exhibit increased levels of circulating TRMs (cTRMs) with origins in the skin, characterized by a pro-inflammatory Th2/Th17 biased activated phenotype. Notably, these cTRMs express gut-homing receptors and are implicated in gastrointestinal GVHD pathogenesis, as evidenced by their presence in intestinal lesions [176]. This suggests that cTRMs can migrate from skin to the gut, precipitating gastrointestinal inflammatory responses.

To date, the potential of disrupting cell trafficking as a treatment for multi-organ comorbidities remains largely unexplored. Vedolizumab, a selective α_4_β_7_ integrin antagonist for IBD treatment [177], has been hypothesized to reduce the severity of cEIMs. This hypothesis rests on the understanding that lymphocytes require the α_4_β_7_–MAdCAM1 interaction for gut access and activation, followed by a non-α_4_β_7_-dependent pathway for skin entry. Yet, there is a lack of substantial real-world research data to support this. Studies, including case series, cohort studies, and randomized controlled trial analyses, suggest that vedolizumab may alleviate skin conditions such as pyoderma gangrenosum, erythema nodosum, or aphthous stomatitis in some IBD patients, but not in all [178–180]. Additionally, vedolizumab has been associated with the onset of new arthritis cases and paradoxical skin lesions [181–183]. These complex phenomena could be due to the drug’s selective inhibition of gut-homing receptors, which might paradoxically lead to an increased accumulation of pathogenic immune cells at extraintestinal sites, or due to the different underlying pathophysiological mechanisms of various cEIMs. A deeper understanding of cell trafficking between the gut and skin is essential to develop innovative therapeutic strategies in vaccine development, immunotherapy, and anti-adhesion therapies.

## Conclusions

The dysregulation of immune cell migration emerges as a contributing factor to the spread of inflammation from primary sites to distant organs. Despite this recognition, critical questions remain unanswered:

1. The specific molecular mechanisms that trigger the reprogramming of homing receptors on immune cells have not been fully characterized.
2. The conditions that precipitate the egress of DCs from local lymph nodes require elucidation.
3. Distinct mechanisms that regulate the recruitment of gut-derived cells to the skin and other extraintestinal locations need to be clarified, highlighting potential differences in these processes [184].

Addressing these fundamental research questions is pivotal for clinical translation. In the realm of clinical applications, research could focus on strategies to induce immune tolerance in the skin to alleviate intestinal inflammation [185] and, conversely, strategies aimed at inducing immune tolerance in the intestines to alleviate cutaneous inflammation. The development of novel vaccination strategies [186, 187] and anti-migratory therapies also holds promise.

## Data Availability

Not applicable.

## Abbreviations

PNAd: Peripheral-node addressin
MAdCAM: Mucosal addressin cell adhesion molecule
ICAM: Intracellular adhesion molecule
VCAM: Vascular cell adhesion molecule
HCAM: Hyaluronate-binding cell adhesion molecule
PSGL: P-selectin glycoprotein ligand
CLA: Cutaneous lymphocyte-associated antigen
VAP: Vascular adhesion protein
PECAM: Platelet-endothelial cell adhesion molecule
JAMs: Junctional adhesion molecules
HEV: High endothelial venule
PP: Peyer’s patch
ILF: Isolated lymphoid follicle
MLN: Mesenteric lymph node
IEL: Intestinal intraepithelial lymphocyte
CCR: C-C chemokine receptor
CXCR: C-X-C chemokine receptor
CCL: C-C motif chemokine ligand
CXCL: C-X-C motif chemokine ligand
AD: Atopic dermatitis
GPR: G protein-coupled receptor
